# Supplementary material for: Altered Functional Connectivity Density in Type 2 Diabetes Mellitus with and without Mild Cognitive Impairment
Source: Brain Sci. 2023 Jan 13;13(1):144. doi: 10.3390/brainsci13010144 (PMC9856282; doi:10.3390/brainsci13010144)
Supplement: Supplementary file 1 [file brainsci-13-00144-s001.zip › brainsci-2050183-supplementary.pdf]

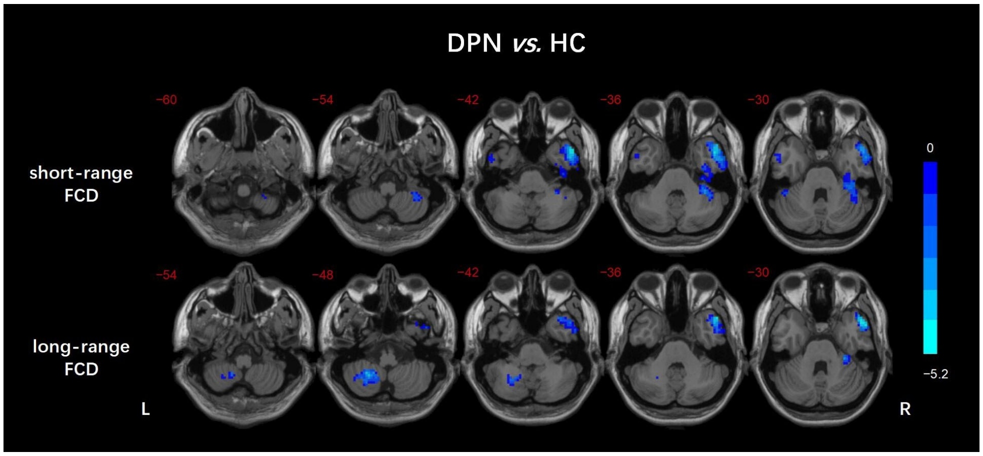

Figure S1. Comparison of short- and long- range FCDs between patients with diabetic peripheral neuropathy (DPN) and HCs (two-sample t-test: GRF corrected $p < 0.005$, cluster level $p < 0.05$). The color scale denotes the t-value. L, left; R, right.
